# Involvement of a flavoprotein, acetohydroxyacid synthase, in growth and riboflavin production in riboflavin-overproducing *Ashbya gossypii* mutant

**DOI:** 10.1186/s12934-023-02114-1

**Published:** 2023-05-22

**Authors:** Tatsuya Kato, Mai Kano, Ami Yokomori, Junya Azegami, Hesham A. El Enshasy, Enoch Y. Park

**Affiliations:** 1 Molecular and Biological Function Research Core, Research Institute of Green Science and Technology, Shizuoka University, Ohya 836, Suruga-Ku, Shizuoka, Japan; 2 Department of Agriculture, Graduate School of Integrated Science and Technology, Shizuoka University, Ohya 836, Suruga-Ku, Shizuoka, Japan; 3 Department of Applied Life Science, Shizuoka University, Ohya 836, Suruga-Ku, Shizuoka, Japan; 4 Institute of Bioproduct Development (IBD), Universiti Teknologi Malaysia (UTM), 81310 UTM Johor Bahru, Malaysia; 5 City of Scientific Research and Technology Applications, New Borg Al Arab, Alexandria Egypt

**Keywords:** *Ashbya gossypii*, Riboflavin, Flavoprotein, Acetohydroxyacid synthase

## Abstract

**Background:**

Previously, we isolated a riboflavin-overproducing *Ashbya gossypii* mutant (MT strain) and discovered some mutations in genes encoding flavoproteins. Here, we analyzed the riboflavin production in the MT strain, in view of flavoproteins, which are localized in the mitochondria.

**Results:**

In the MT strain, mitochondrial membrane potential was decreased compared with that in the wild type (WT) strain, resulting in increased reactive oxygen species. Additionally, diphenyleneiodonium (DPI), a universal flavoprotein inhibitor, inhibited riboflavin production in the WT and MT strains at 50 µM, indicating that some flavoproteins may be involved in riboflavin production. The specific activities of NADH and succinate dehydrogenases were significantly reduced in the MT strain, but those of glutathione reductase and acetohydroxyacid synthase were increased by 4.9- and 25-fold, respectively. By contrast, the expression of *AgGLR1* gene encoding glutathione reductase was increased by 32-fold in the MT strain. However, that of *AgILV2* gene encoding the catalytic subunit of acetohydroxyacid synthase was increased by only 2.1-fold. These results suggest that in the MT strain, acetohydroxyacid synthase, which catalyzes the first reaction of branched-chain amino acid biosynthesis, is vital for riboflavin production. The addition of valine, which is a feedback inhibitor of acetohydroxyacid synthase, to a minimal medium inhibited the growth of the MT strain and its riboflavin production. In addition, the addition of branched-chain amino acids enhanced the growth and riboflavin production in the MT strain.

**Conclusion:**

The significance of branched-chain amino acids for riboflavin production in *A. gossypii* is reported and this study opens a novel approach for the effective production of riboflavin in *A. gossypii*.

**Supplementary Information:**

The online version contains supplementary material available at 10.1186/s12934-023-02114-1.

## Background

*Ashbya gossypii* yields riboflavin and has been used for industrial riboflavin production [1, 2]. Riboflavin is a precursor of flavin adenine dinucleotide (FAD) and flavin mononucleotide (FMN), which are cofactors of flavoproteins. Humans cannot produce riboflavin because they lack the riboflavin biosynthetic pathway. Thus, some diseases are caused by riboflavin deficiency [3].

Riboflavin is required for the function of flavoproteins, which are involved in different cell responses [4]. Riboflavin is associated with the functions of the mitochondria, which contain several flavoproteins. For example, complexes I and II in the electron transport chain (ETC) retain FMN and FAD, and electron transfer flavoprotein (ETF) and ETF-ubiquinone oxidoreductase (ETF-QO), which transfer an electron to ubiquinone in the β-oxidation of fatty acids in the mitochondria, are also flavoproteins. Additionally, riboflavin has vital functions in maintaining cellular redox balance through the glutathione redox cycle [5].

To date, several metabolic analyses and engineering studies have been performed to enhance riboflavin production [6, 7]. The riboflavin biosynthetic pathway and purine biosynthetic pathway are both important for riboflavin production, and glycine is known as an additive to help with riboflavin production. Additionally, oxidative stress and DNA damage are connected with the overproduction of riboflavin in *A. gossypii* [8–10].

In *A. gossypii*, the association of riboflavin with flavoprotein functions has never been reported. In our previous report, some mutations in genes encoding flavoproteins were discovered in the riboflavin-overproducing mutant isolated by disparity mutagenesis [11, 12]. It implies that the activity of flavoproteins may be changed in this riboflavin-overproducing mutant. In this study, we investigated the activity of flavoprotein in this mutant and the association of flavoproteins with riboflavin production.

## Results

### Mutations of flavoprotein genes in the riboflavin-overproducing mutant

**Fig. 1:**
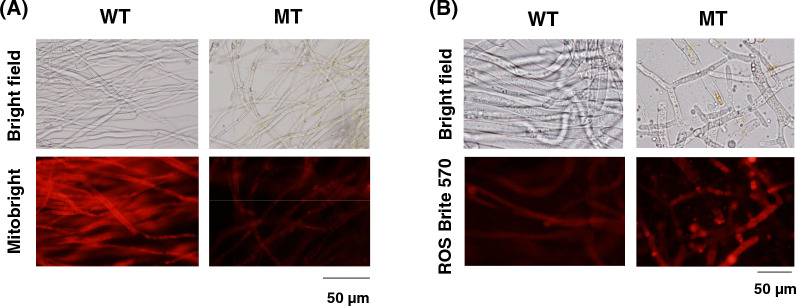
Mitochondrial membrane potential and ROS in WT and MT strains. **A** Staining of mitochondrial membrane potential in WT and MT strains. Mitochondrial membrane potential was stained with MitoBright LT in WT and MT strains as per the protocol described in “Methods” section. **B** Staining of ROS in WT and MT strains. ROS was stained with ROS Brite in WT and MT strains as per the protocol described in “Methods” section

Our previous study discovered 33 homokaryotic and 1377 heterokaryotic mutations in a riboflavin-overproducing mutant isolated by disparity mutagenesis [11]. In particular, genes encoding flavoproteins in the mitochondria have these mutations. These results suggest that the function of the mitochondria in the MT strain may be changed. Mitochondrial membrane potential was stained with MitoBright LT Red to prove this (Fig. 1A). Faint fluorescence was observed in the MT strain compared to that in the WT strain. This result indicates that the dysfunction of the mitochondria in the MT strain occurs. The decrease in mitochondrial membrane potential is linked with ROS production [13, 14]. Subsequently, ROS was stained with ROS Brite 570 in both strains (Fig. 1B). Hydroxy radicals and superoxides were also stained with ROS Brite 570. An increase in fluorescence in the MT strain was observed compared with that of the WT strain. This result indicates that the MT strain produced hydroxy radicals and superoxides parallel to mitochondrial dysfunction.

**Fig. 2:**
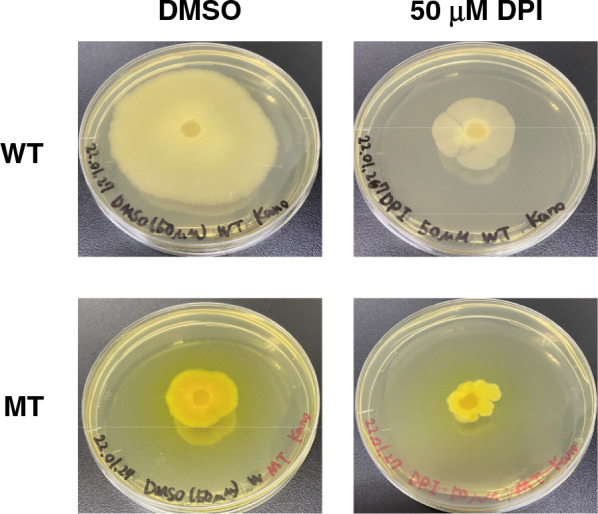
The effect of DPI on riboflavin production in WT and MT strains. Both WT and MT strains were cultivated in YD medium in the presence of 50 µM DPI for 6 days

Diphenyleneiodonium (DPI) is a flavoprotein inhibitor [15]. WT and MT strains were cultivated in the presence of 50 µM DPI to investigate the contribution of flavoproteins to riboflavin production (Fig. 2). In both strains, the yellowing of the mycelia diminished. This result indicates that DPI inhibited riboflavin production in both strains and that flavoproteins may be involved in producing riboflavin in *A. gossypii*.

### Activities of flavoproteins in the MT strain

**Fig. 3:**
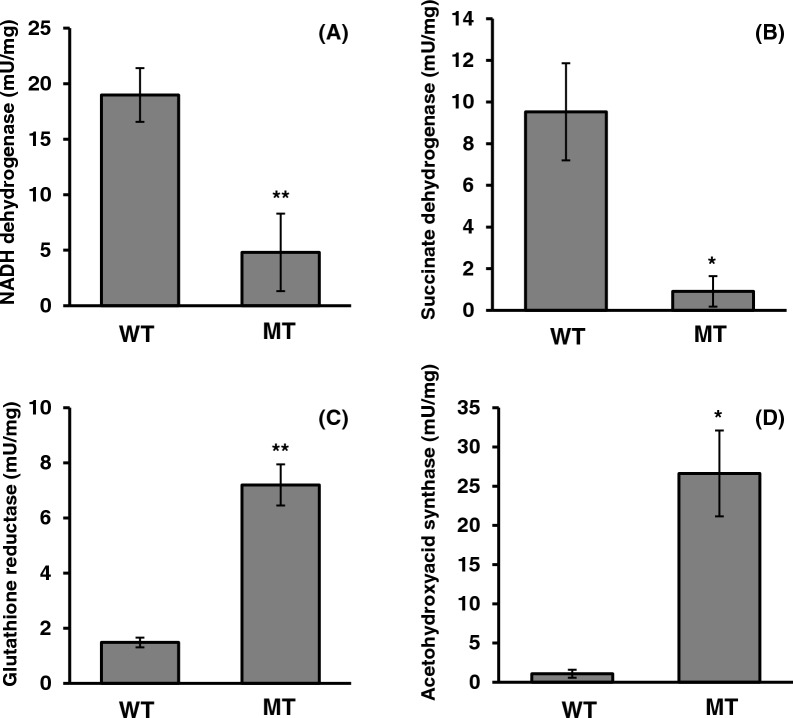
Enzymatic activities of flavoproteins in WT and MT strains: **A** NADH dehydrogenase, **B** succinate dehydrogenase, **C** glutathione reductase, and **D** acetohydroxyacid synthase. Each strain was cultivated in YD liquid medium for a day, and mycelia were collected. Mycelia were suspended in PBS, and the homogenate was prepared by sonication. Each enzymatic activity and protein concentration were determined according to the protocol described in “Methods” section. The error bars represent the standard deviation (n = 3). *p < 0.05, **p < 0.01

Some flavoprotein activities in the MT strain were measured to reveal the involvement of flavoproteins in riboflavin production. Most of the flavoproteins are localized in the mitochondria. In Fig. 1A, mitochondrial membrane potential was decreased in the MT strain. Furthermore, the *AgNDI1* gene, which encodes NADH dehydrogenase, and the *AgSDH1* gene, which encodes a subunit of succinate dehydrogenase, have heterokaryotic mutations in the MT strain (Table 1) [11]. Regarding succinate dehydrogenase, *AgSDH2* and *AgSDH3* genes also have heterokaryotic mutations in the MT strain. NADH dehydrogenase and succinate dehydrogenase are in ETC. Therefore, we first measured NADH dehydrogenase and succinate dehydrogenase activities in both strains. Additionally, the activities of two mitochondrial flavoproteins, namely, glutathione reductase and acetohydroxyacid synthase, were measured because the *AgGLR1* gene encoding glutathione reductase and *AgILV2* gene encoding acetohydroxyacid synthase also have mutations in the MT strain (Table 1). The specific activities of NADH dehydrogenase and succinate dehydrogenase in the MT strain were significantly lower than that in the WT strain (Fig. 3). However, the specific activities of glutathione reductase and acetohydroxyacid synthase were increased by approximately 4.9- and 25-fold, respectively. The MT strain was isolated in the presence of H_2_O_2_ many times during disparity mutagenesis [12]. This increased glutathione reductase activity in the MT strain could be attributed to its isolation in the presence of H_2_O_2_.

**Table 1:** Mutations in genes of MT strain

Gene	Product	DNA changes	Protein changes	Karyotic
AGOS_AAL021W	Small subunit of acetohydroxyacid synthase (AgILV6)	c.140G > A	p.S47N	Hetero
AGOS_AAL021W	Small subunit of acetohydroxyacid synthase (AgILV6)	c.155G > A	p.S52N	Hetero
AGOS_AAL021W	Small subunit of acetohydroxyacid synthase (AgILV6)	c.673G > T	p.G225C	Hetero
AGOS_ACR052W	Flavoprotein subunit of succinate dehydrogenase (AgSDH1)	c.1132G > A	p.D378N	Hetero
AGOS_ACL065C	Iron-sulfur protein subunit of succinate dehydrogenase (AgSDH2)	c.224C > T	p.T75M	Hetero
AGOS_ACL065C	Iron-sulfur protein subunit of succinate dehydrogenase (AgSDH2)	c.697A > C	p.T233P	Hetero
AGOS_AEL305C	Acetohydroxyacid synthase (AgILV2)	c. 1365G > T	p.Q455H	Homo
AGOS_AFR447C	NADH:ubiquinone oxidoreductase (AgNDI1)	c.943G > A	p.V315M	Hetero
AGOS_AGR196W	Glutathione-disulfide reductase (AgGLR1)	c.1415C > A	p.S472Y	Hetero

**Fig. 4:**
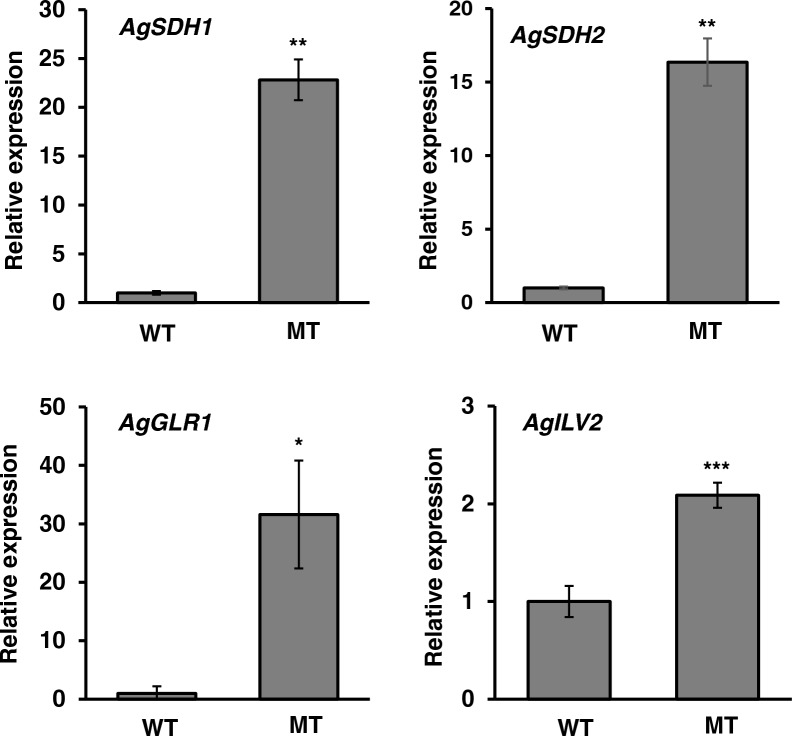
Expression of genes encoding flavoproteins in WT and MT strains. Total RNA was extracted from the mycelia cultivated in YD liquid medium for a day. RT-qPCR was performed after cDNA preparation as per the protocol described in “Methods” section. The error bars represent the standard deviation (n = 3). *p < 0.05, **p < 0.01, ***p < 0.001

RT-qPCR also investigated the expression level of each gene to confirm whether the increase in the specific activities of glutathione reductase and acetohydroxyacid synthase is caused by gene expression (Fig. 4). All genes were expressed more highly in the MT strain than in the WT strain, except for the *AgILV2* gene, which was expressed only 2.3-fold more highly in the MT strain than in the WT strain. In particular, the expression of the *AgGLR1* gene was significantly higher in the MT strain. The activity of glutathione reductase was approximately 4.9 times higher in the MT strain than in the WT strain, and the expression level of the *AgGLR1* gene was approximately 32 times higher than that in the WT strain. These results imply that the specific activity of mutated glutathione reductase may be lower than that of a native form, even though its activity was higher in the MT strain than in the WT strain. In the case of acetohydroxyacid synthase, the expression of the *AgILV2* gene was 2.3-fold higher in the MT strain than in the WT strain, and the activity of acetohydroxyacid synthase was 25-fold higher in the MT strain than in the WT strain. These results imply that the specific activity of mutated acetohydroxyacid synthase might be higher than that of the WT.

### Effects of branched-chain amino acids on the growth and riboflavin production in MT strain

**Fig. 5:**
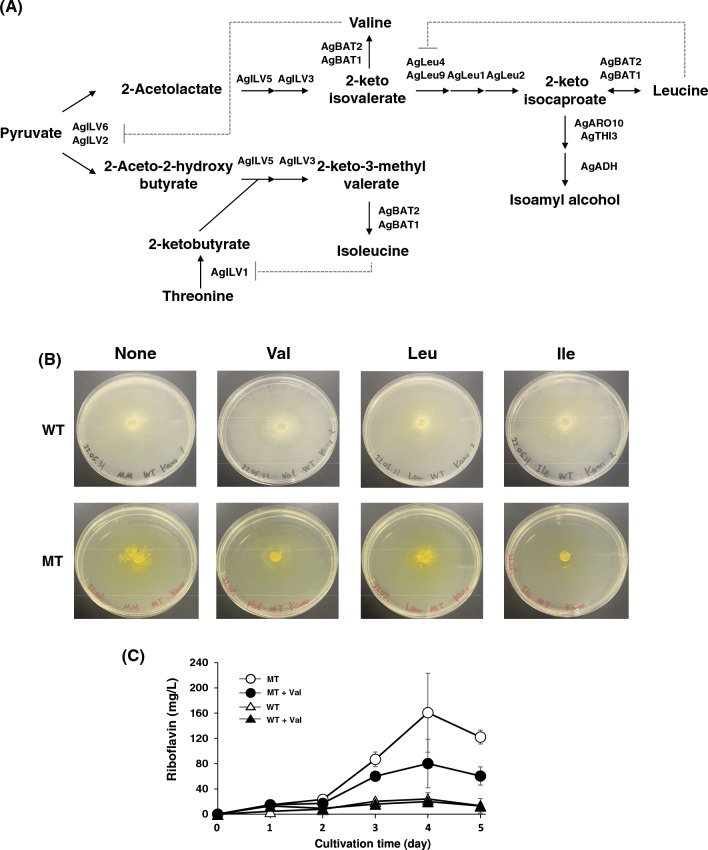
Effects of branched-chain amino acids on the growth and the riboflavin production of WT and MT strains. **A** Pathway of branched-chain amino acid biosynthesis. Dot lines indicate the feedback inhibition of each amino acid. **B** Growth and mycelial color of WT and MT strains in minimal agar medium with each branched-chain amino acid. WT and MT strains were cultivated in minimal agar medium with 1 mM each branched-chain amino acid for 2 weeks. **C** Time course of riboflavin production in WT and MT strains in minimal liquid medium in the presence of valine. WT and MT strains were cultivated in the minimal liquid medium with 1 mM valine for 5 days, and the production of riboflavin was measured. The error bars represent the standard deviation (n = 3)

Acetohydroxyacid synthase catalyzes the first reaction in the biosynthesis of branched-chain amino acids. *ILV2* gene encodes a catalytic subunit of yeast acetohydroxyacid synthase, which is regulated by *ILV6* gene encoded regulatory subunit [16]. In *A. gossypii*, this enzyme is composed of AgILV2 and AgILV6 subunits. The disruption of the *AgILV2* gene was performed to investigate the effect of acetohydroxyacid synthase on riboflavin production in *A. gossypii*. However, we could not isolate the homokaryotic *AgILV2* gene-disrupted mutant based on the WT and MT strains. Therefore, the inhibition of acetohydroxyacid synthase by valine was conducted. Valine inhibits the activity of the ILV2–ILV6 complex by feedback inhibition (Fig. 5A) [16, 17]. Therefore, both strains of *A. gossypii* were grown in minimal medium with branched-chain amino acids to inhibit acetohydroxyacid synthase (Fig. 5B). The growth of the WT strain was observed in minimal medium and in the presence of valine, leucine, and isoleucine. However, valine slightly inhibited the growth of the WT strain and made the mycelia white, suggesting that valine inhibits riboflavin production. Although the growth of the MT strain was also seen in the minimal medium, it was inhibited by the presence of valine (Fig. 5B). Valine inhibits the yeast acetohydroxyacid synthase composed of ILV2 and ILV6 [16]. These results imply that valine may greatly influence the growth of the MT strain because valine may inhibit acetohydroxyacid synthase in the MT strain. Therefore, acetohydroxyacid synthase may be important for the growth of the MT strain. The amount of riboflavin produced by the MT strain in the minimal liquid medium supplemented with 1 mM valine was lower by approximately 50% than that in the medium without valine (Fig. 5C). This result showed that acetohydroxyacid synthase is important for riboflavin production in the MT strain. However, because the riboflavin production in the WT strain was very low, we could not distinguish between the differences in riboflavin production regardless of valine.

Isoleucine suppressed the growth of the MT strain and valine (Fig. 5B). Furthermore, isoleucine inhibits threonine deaminase encoded by the *ILV1* gene in *Saccharomyces cerevisiae* (Fig. 5A) [18] and represses acetohydroxyacid synthase in a minimal medium [19]. These results imply that the addition of isoleucine may also suppress threonine deaminase or/and acetohydroxyacid synthase activity, resulting in the growth deficiency of the MT strain.

**Fig. 6:**
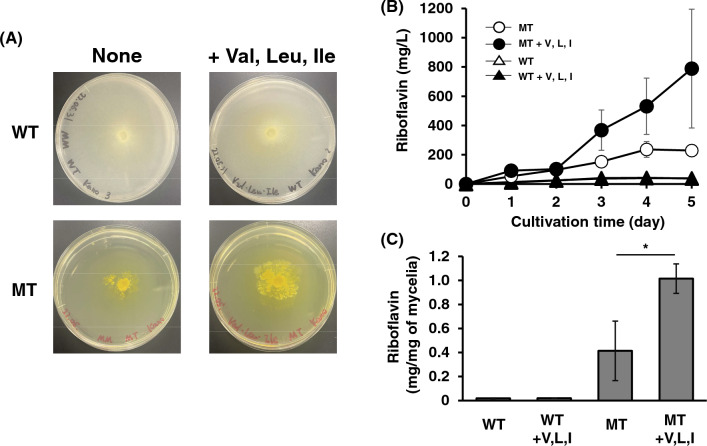
Effects of three branched-chain amino acids on the riboflavin production of WT and MT strains. **A** Growth and mycelial color of WT and MT strains in minimal agar medium with three branched-chain amino acids. WT and MT strains were cultivated in minimal agar medium with three branched-chain amino acids (1 mM each) for 2 weeks. **B** Time course of the riboflavin production in WT and MT strains in minimal liquid medium with three branched-chain amino acids at 1 mM each. WT and MT strains were cultivated in the minimal liquid medium with 1 mM valine for 5 days, and the production of riboflavin was measured. The error bars represent the standard deviation (n = 3). **C** Specific riboflavin production of the MT strain in minimal liquid medium with three branched-chain amino acids at 1 mM each. MT strain was cultivated for 9 days. Riboflavin concentration and dry mycelial weight were measured. The error bars represent the standard deviation (n = 3). *p < 0.05

Additionally, the addition of three branched-chain amino acids allowed the mycelia of WT and MT strains to become yellow in the minimal agar medium (Fig. 6). The growth of the MT strain was greatly enhanced by three branched-chain amino acids (Fig. 6A). In the minimum liquid medium, the specific riboflavin production of the MT strain with three branched-chain amino acids was 2.5 times higher than that without these amino acids (Fig. 6B and C). These results imply that branched amino acids may be also involved in the growth and riboflavin production in the MT strain. However, we were not also able to detect the differences in riboflavin production in the WT in the presence of three branched-chain amino acids because the riboflavin production in the WT strain was very low.

## Discussion

ROS production in the MT strain was increased compared with that in the WT strain (Fig. 1). ROS production is involved in riboflavin production in *A. gossypii* [10]. Higher amount of ROS in the MT strain corresponds to that in a previous report [10]. Mitochondrial dysfunction produces ROS in *S. cerevisiae* [20, 21]. In this study, the mitochondrial membrane potential in the MT strain was decreased compared with that in the WT strain and the activities of NADH dehydrogenase and succinate dehydrogenase in the MT strain were significantly reduced compared to those in the WT strain (Figs. 1 and 3). These findings imply that the mitochondria in the MT strain may be dysfunctional. The mitochondria are sources of ROS, and increased ROS induces the activation of antioxidant systems in the mitochondria [20, 22]. The results in this study contradict the earlier finding that the expression of the *AgSDH1* gene, which codes for a succinate dehydrogenase subunit, was lower in the MT strain than in the WT strain [12]. In this study, the MT strain was grown for 1 day in YD medium with glucose as the sole carbon source, whereas in the previous study, the MT strain was grown for 4 days in YR medium based on rapeseed oil as the sole carbon source. In yeast and *Escherichia coli*, genes encoding subunits of succinate dehydrogenase are repressed by glucose [23, 24]. Conversely, the expression of these genes in *Kluyveromyces lactis* are not repressed by glucose [25]. A carbon source in the culture medium also influences the expression of these genes in *A. gossypii*, which might have caused the inconsistency between this study and the previous study.

The activity of glutathione reductase encoded by *AgGLR1* was higher in the MT strain than in the WT strain (Fig. 3). Glutathione reductase is a flavoprotein and needs FAD for its activity. DPI, which is an inhibitor of flavoproteins, reduced the production of riboflavin in the MT strain (Fig. 2). However, 50 µM 2-acetylamino-3-[4-(2-acetylamino-2-carboxyethylsulfanylthiocarbonylamino) phenylthiocarbamoylsulfanyl] propionic acid (2-AAPA), which is an inhibitor of glutathione reductase, did not have any effects on the mycelial color of the WT and MT strains (Additional file 1). These findings demonstrate that glutathione reductase is not involved in riboflavin production in *A. gossypii*, even though it is a flavoprotein.

In addition to glutathione reductase, the activity of acetohydroxyacid synthase, a flavoprotein that catalyzes the first reaction in synthesizing branched-chain amino acids from pyruvate, was higher in the MT strain than in the WT strain (Fig. 3). The inhibitor of acetohydroxyacid synthase, valine, and isoleucine inhibited the growth of the MT strain (Fig. 5A). Additionally, three branched-chain amino acids enhanced the growth and the riboflavin production in the MT strain (Fig. 6). *A. gossypii* produces a large amount of isoamyl alcohol synthesized by the degradation of leucine or from an intermediate in the leucine biosynthesis pathway, α-ketoisocaproate (Fig. 5A) [26, 27]. These findings imply that *A. gossypii* may primarily produce and use leucine and other branched-chain amino acids as a nutrient for the growth. However, the relationship of leucine with riboflavin production is yet to be revealed. Acetohydroxyacid synthase needs NAD and FAD as cofactors for its activity [28]. Furthermore, in yeast, acetohydroxyacid synthase is regulated by redox conditions through menadione, suggesting that redox conditions may control branched-chain amino acid synthesis [29]. In the MT strain, a higher amount of ROS was observed compared with that in the WT strain (Fig. 1), and other riboflavin-overproducing mutants produce higher ROS [10]. In the *Bacillus subtilis pta*-disrupted mutant, the overexpression of acetohydroxyacid synthase leads to riboflavin production enhancement [30]. In this study, we did not examine the direct relationship between riboflavin production with acetohydroxyacid synthase and branched-chain amino acid biosynthesis. However, three branched-chain amino acids enhanced the growth and the riboflavin production in the MT strains (Fig. 6). The riboflavin-overproducing MT strain has a higher activity of acetohydroxyacid synthase, a flavoprotein crucial for the growth of the MT strain, compared to the WT strain. This study suggests that acetohydroxyacid synthase may be a target for the riboflavin overproduction in *A. gossypii* and that branched amino acids are important factors for riboflavin production in *A. gossypii*.

## Conclusion

The riboflavin-overproducing *A. gossypii* mutant (MT strain), which was previously isolated by disparity mutagenesis, produced higher amount of ROS than the WT strain and mitochondria of the MT strain was compromised. The specific activity of acetohydroxyacid synthase, which is a flavoprotein in mitochondria and catalyzes the first reaction in the branched-chain amino acid biosynthesis pathway, in the MT strain was 25-fold higher than that of the WT strain. Valine, which is known as a feedback inhibitor, suppressed the growth and riboflavin production in the MT strain at 1 mM, but the addition of three branched-chain amino acids enhanced the growth and riboflavin production in the MT strain at 1 mM each. These results suggest that branched-amino acids are important for the growth and riboflavin production in the MT strain and lead to the creation of the new strategy of the metabolic engineering for the riboflavin overproduction in *A. gossypii*.

## Methods

### Strains and cultivation

The wild type (WT) strain used was *A. gossypii* ATCC10895. Our previous study isolated a riboflavin-overproducing mutant (MT) strain by disparity mutagenesis [12]. These strains were cultivated on YD agar medium (1% glucose, 1% yeast extract, pH 6.8) at 27 ℃. For liquid cultivation, both strains were grown in 10 mL YD medium at 27 ℃ for 24 h, followed by cultivation in 30-mL YD medium at 27 ℃ for 24 h as precultivation. Subsequently, both strains were cultivated in 50 mL YD medium at 27 ℃ for indicated time. Cultivation of *A. gossypii* in the minimal medium was performed according to the previous study [31].

### Detection of mitochondrial membrane potential and reactive oxygen species (ROS)

Mitochondrial membrane potential was stained with MitoBright LT Red (Dojindo, Kumamoto, Japan) as per the attached protocol. First, mycelia were incubated with 0.1 µM MitoBright LT Red at 27 ℃ for 15 min. Subsequently, the supernatant was removed after centrifugation, and the mycelia were washed with phosphate-buffered saline (PBS, pH 7.4) three times. Fluorescence in both strains was observed under fluorescent microscope BX60 (Olympus, Tokyo, Japan).

ROS in mycelia were stained with ROS Brite 570 (AAT Bioquest, Sunnyvale, CA, USA) following the attached protocol. Mycelia were incubated with 5 µM ROS Brite 570 at 27 ℃ for 30 min. The supernatant was removed after centrifugation, and mycelia were washed three times with PBS (pH 7.4). Fluorescence was observed in both strains under fluorescence microscope BX60.

### Assays of each flavoprotein

Mycelia cultivated in YD liquid medium for 24 h were obtained by filtration and suspended in PBS. Mycelia were disrupted by sonication, and the cell lysate was used for these assays as described below.

As described previously, NADH dehydrogenase activity was also measured [32]. Briefly, the reaction mixture (990 µL) containing 0.05 mM 2,6-dichloroindophenol sodium (DCIP), 0.1 mM NADH, 200 mM KCl, and 1 mM EDTA in 0.1 M potassium phosphate (pH 6.0) was preincubated at room temperature. To start the reaction, 10 µL of the crude extract was added to the reaction mixture. The extinction coefficient of reduced DCIP was used to calculate the slope of the absorbance at 600 nm (1.9 × 10^4^ M^−1^ cm^−1^).

Succinate dehydrogenase activity was estimated as previously described [33]. Briefly, the reaction mixture (490 µL) containing 0.1% 3-(4,5-dimethylthiazol-2-yl)-2,5-diphenyltetrazolium bromide (MTT) and 50 mM sodium succinate in PBS was incubated at 30 °C as preincubation, and 10 µL of the crude extract was added into the reaction mixture to start the reaction. Next, 1 mL of acetone was added to the reaction mixture, and the mixture was stirred completely after 10 min. Absorbance in the acetone fraction was measured at 570 nm after centrifugation.

Glutathione reductase activity was measured using an OxiSelect Glutathione Reductase Assay Kit (Cell Biolabs, San Diego, CA, USA) following the attached protocol. Briefly, 100 µL of cell lysate was mixed with 25 µL of 1 × NADPH solution and 1 × chromogen in a well of a 96-well plate, and the 96-well plates were set to a filter-based multimode microplate reader (Infinite F200 M). Subsequently, 25 mL of glutathione disulfide (GSSH) was added, and the absorbance increase at 405 nm was monitored.

The activity of acetohydroxyacid synthase was measured as previously reported [16]. The reaction mixture (450 µL) containing 50 mM pyruvate, 1 mM thiamin diphosphate, 10 mM MgCl_2_, and 10 µM FAD in 50 mM potassium phosphate buffer (pH 7.0) was incubated at 30 °C as preincubation, and the reaction was started by the addition of 50 µL of cell lysate. After the incubation for 3 h, the reaction was stopped by the addition of 50 µL of 5% H_2_SO_4_. The mixture was incubated at 60 °C for 30 min to convert 2-acetolactate to acetoin. Then, 500 µL of 0.5% creatine and 500 µL of 5% α-naphthol in 2.5 N NaOH were added to the reaction mixture, and the mixture was incubated at 37 °C for 30 min to quantify the produced acetoin. The absorbance was at 525 nm.

One unit of activity in each assay was defined as the amount of enzyme needed to produce 1 µmol of an enzymatic product in 1 min. Protein concentration was determined using a Pierce BCA Protein Assay Kit (Thermo Fisher Scientific K.K., Tokyo, Japan).

### RT-qPCR

Total RNA was extracted from mycelia with Trizol (Thermo Fisher Scientific K.K., Tokyo, Japan). Mycelia (100 mg) were placed into liquid nitrogen and incubated for 1 min. Frozen mycelia were crashed, and 1 mL Trizol was added to the sample. After incubation at room temperature for 5 min, 200 µL chloroform was added to the mixture. The mixture was incubated for 3 min before being centrifuged at 12,000*g* for 15 min, and the supernatant was collected. Following that, 2-propanol precipitation was performed. The precipitated RNA was dissolved in RNA-free water before being treated with DNase and purified.

PrimeScript RT-PCR Kit (Takara Bio, Kusatsu, Japan) and 500 ng of total RNA were used to prepare cDNA. Quantitative PCR was performed using Thunderbird SYBR qPCR Mix (Toyobo, Osaka, Japan). Data were analyzed by the comparative CT method (2^∆∆CT^ method) using the *AgTEF1* gene (AGOS_ADL370C) of *A. gossypii*. The sequences of the primers are presented in Table 2.

**Table 2:** Primers used in this study

Primer	5′–3′
AgSDH1-F	tctgccgctgagagaaaaga
AgSDH1-R	agtaagcacgtacggttgga
AgSDH2-F	agtcgatccagccatacctg
AgSDH2-R	gctcgttgttccaccagtac
AgGLR1-F	acttggttattggaggcgga
AgGLR1-R	tgctgatgtcgacttcctgt
AgILV2-F	gcatcgcggctcatcaatat
AgILV2-R	gcccagcatatctacggact

## Supplementary Information

**Additional file 1.** Effects of 2-AAPA on the growth and the riboflavin production of the WT strain. The WT strain was cultivated in the presence of 10 μM 2-AAPA on YD agar medium for 6 days.

## Data Availability

Data in this manuscript can be shared on request to the corresponding author.

## References

[1] Revuelta JL, Ledesma-Amaro R, Lozano-Martinez P, Díaz-Fernández D, Buey RM, Jiménez A (2017). Bioproduction of riboflavin: a bright yellow history. J Ind Microbiol Biotechnol.

[2] You J, Pan X, Yang C, Du Y, Osire T, Yang T, Zhang X, Xu M, Xu G, Rao Z (2021). Microbial production of riboflavin: biotechnological advances and perspectives. Metab Eng.

[3] Mosegaard S, Dipace G, Bross P, Carlsen J, Gregersen N, Olsen RKJ (2020). Riboflavin deficiency-Implications for general human health and inborn errors of metabolism. Int J Mol Sci.

[4] Suwannasom N, Kao I, Pruß A, Georgieva R, Bäumler H (2020). Riboflavin: the health benefits of a forgotten natural vitamin. Int J Mol Sci.

[5] Ashoori M, Saedisomeolia AA (2014). Riboflavin (vitamin B_2_) and oxidative stress: a review. Br J Nutr.

[6] Schwechheimer SK, Park EY, Revuelta JL, Becker J, Wittmann C (2016). Biotechnology of riboflavin. Appl Microbiol Biotechnol.

[7] Zhao G, Dong F, Lao X, Zheng H (2021). Strategies to increase the production of biosynthetic riboflavin. Mol Biotechnol.

[8] Kato T, Azegami J, Kano M, El Enshasy HA, Park EY (2021). Effects of sirtuins on the riboflavin production in *Ashbya gossypii*. Appl Microbiol Biotechnol.

[9] Kavitha S, Chandra TS (2014). Oxidative stress protection and glutathione metabolism in response to hydrogen peroxide and menadione in riboflavinogenic fungus *Ashbya gossypii*. Appl Biochem Biotechnol.

[10] Silva R, Aguiar TQ, Oliveira R, Domingues L (2019). Light exposure during growth increases riboflavin production, reactive oxygen species accumulation and DNA damage in *Ashbya gossypii* riboflavin-overproducing strains. FEMS Yeast Res..

[11] Kato T, Azegami J, Yokomori A, Dohra H, El Enshasy HA, Park EY (2020). Genomic analysis of a riboflavin-overproducing *Ashbya gossypii* mutant isolated by disparity mutagenesis. BMC Genomics..

[12] Park EY, Ito Y, Nariyama M, Sugimoto T, Lies D, Kato T (2011). The improvement of riboflavin production in *Ashbya gossypii* via disparity mutagenesis and DNA microarray analysis. Appl Microbiol Biotechnol..

[13] Bhatti JS, Bhatti GK, Reddy PH (2017). Mitochondrial dysfunction and oxidative stress in metabolic disorders: a step towards mitochondria based therapeutic strategies. Biochim Biophys Acta Mol Basis Dis.

[14] Pieczenik SR, Neustadt J (2007). Mitochondrial dysfunction and molecular pathways of disease. Exp Mol Pathol.

[15] Ratz JD, McGuire JJ, Anderson DJ, Bennett BM (2000). Effects of the flavoprotein inhibitor, diphenyleneiodonium sulfate, on ex vivo organic nitrate tolerance in the rat. J Pharmacol Exp Ther.

[16] Pang SS, Duggleby RG (1999). Expression, purification, characterization, and reconstitution of the large and small subunits of yeast acetohydroxyacid synthase. Biochemistry.

[17] Pang SS, Duggleby RG (2001). Regulation of yeast acetohydroxyacid synthase by valine and ATP. Biochem J.

[18] Isogai S, Nishimura A, Kotaka A, Murakami N, Hotta N, Ishida H, Takagi H (2022). High-level production of isoleucine and fusel alcohol by expression of the feedback inhibition-insensitive threonine deaminase in *Saccharomyces cerevisiae*. Appl Environ Microbiol..

[19] Bollon AP, Magee PT (1971). Involvement of threonine deaminase in multivalent repression of the isoleucine-valine pathway in *Saccharomyces cerevisiae*. Proc Natl Acad Sci USA.

[20] Knorre DA, Sokolov SS, Zyrina AN, Severin FF (2016). How do yeast sense mitochondrial dysfunction?. Microb Cell..

[21] Yi DG, Hong S, Huh WK (2018). Mitochondrial dysfunction reduces yeast replicative lifespan by elevating RAS-dependent ROS production by the ER-localized NADPH oxidase Yno1. PLoS ONE..

[22] Zuin A, Gabrielli N, Calvo IA, García-Santamarina S, Hoe KL, Kim DU, Park HO, Hayles J, Ayté J, Hidalgo E (2008). Mitochondrial dysfunction increases oxidative stress and decreases chronological life span in fission yeast. PLoS One..

[23] Lombardo A, Carine K, Scheffler IE (1990). Cloning and characterization of the iron-sulfur subunit gene of succinate dehydrogenase from *Saccharomyces cerevisiae*. J Biol Chem.

[24] Nam TW, Park YH, Jeong HJ, Ryu S, Seok YJ (2005). Glucose repression of the Escherichia coli sdhCDAB operon, revisited: regulation by the CRP•cAMP complex. Nucleic Acids Res.

[25] Saliola M, Bartoccioni PC, De Maria I, Lodi T, Falcone C (2004). The deletion of the succinate dehydrogenase gene KlSDH1 in *Kluyveromyces lactis* does not lead to respiratory deficiency. Eukaryot Cell.

[26] Holt S, Miks MH, de Carvalho BT, Foulquié-Moreno MR, Thevelein JM (2019). The molecular biology of fruity and floral aromas in beer and other alcoholic beverages. FEMS Microbiol Rev.

[27] Ravasio D, Wendland J, Walther A (2014). Major contribution of the Ehrlich pathway for 2-phenylethanol/rose flavor production in *Ashbya gossypii*. FEMS Yeast Res.

[28] Liu Y, Li Y, Wang X (2016). Acetohydroxyacid synthases: evolution, structure, and function. Appl Microbiol Biotechnol.

[29] Lonhienne T, Garcia MD, Guddat LW (2017). The role of a FAD cofactor in the regulation of acetohydroxyacid synthase by redox signaling molecules. J Biol Chem..

[30] Zhu Y, Chen X, Chen T, Zhao X (2007). Enhancement of riboflavin production by overexpression of acetolactate synthase in a *pta* mutant of *Bacillus subtilis*. FEMS Microbiol Lett.

[31] Jeong BY, Wittmann C, Kato T, Park EY (2013). Comparative metabolic flux analysis of an *Ashbya gossypii* wild type strain and a high riboflavin-producing mutant strain. J Biosci Bioeng.

[32] Velázquez I, Pardo JP (2001). Kinetic characterization of the rotenone-insensitive internal NADH: ubiquinone oxidoreductase of mitochondria from *Saccharomyces cerevisiae*. Arch Biochem Biophys.

[33] Munakata H (1993). Fundamental and clinical studies of chemosensitivity test performed by measuring specific activity of succinate dehydrogenase in subrenal capsule assay—influence of host immune response and clinical correlation-. Jpn J Oral Maxillofac Surg.

