# Supplementary figures and images for: Involvement of a flavoprotein, acetohydroxyacid synthase, in growth and riboflavin production in riboflavin-overproducing Ashbya gossypii mutant
Source: Microb Cell Fact. 2023 May 22;22:105. doi: 10.1186/s12934-023-02114-1 (PMC10201721; doi:10.1186/s12934-023-02114-1)

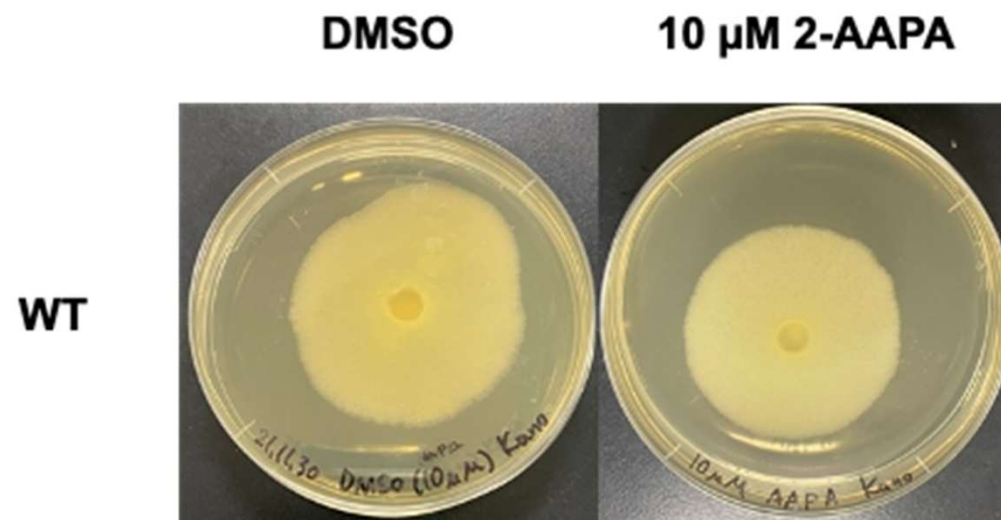

Supplement: Supplementary file 1 — Additional file 1. Effects of 2-AAPA on the growth and the riboflavin production of the WT strain. The WT strain was cultivated in the presence of 10 μM 2-AAPA on YD agar medium for 6 days. [file 12934_2023_2114_MOESM1_ESM.pdf]
